# Supplementary figures and images for: A conserved role of bam in maintaining metabolic homeostasis via regulating intestinal microbiota in Drosophila
Source: PeerJ. 2022 Oct 10;10:e14145. doi: 10.7717/peerj.14145 (PMC9559046; doi:10.7717/peerj.14145)

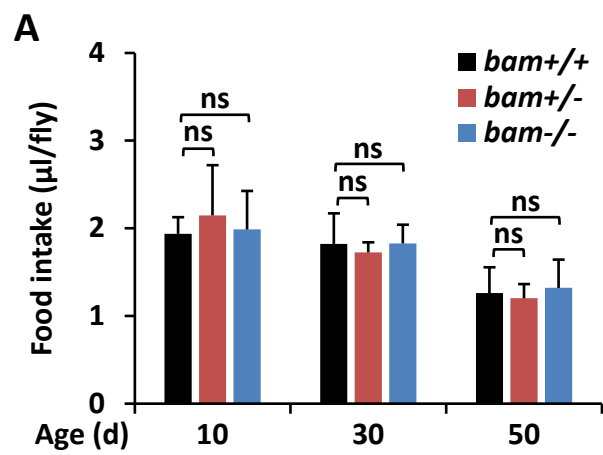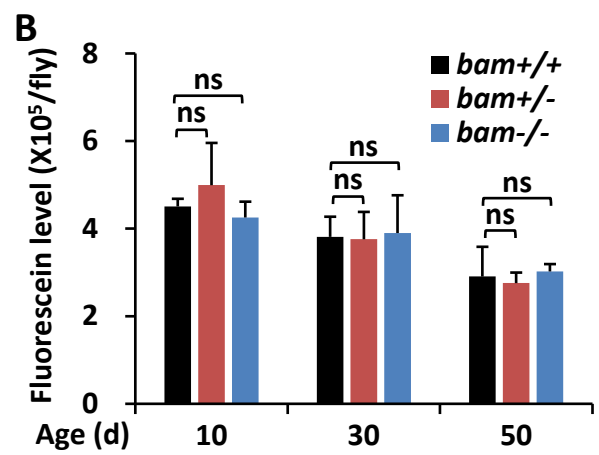

Supplement: Figure S1 — (A) Cafe assay. (B) Fluorescein feeding assay. Error bars represent SD (n = 3). The two-tailed Student’s t test was used to analyze statistical significance. ns, not significant. [file peerj-10-14145-s001.pdf]

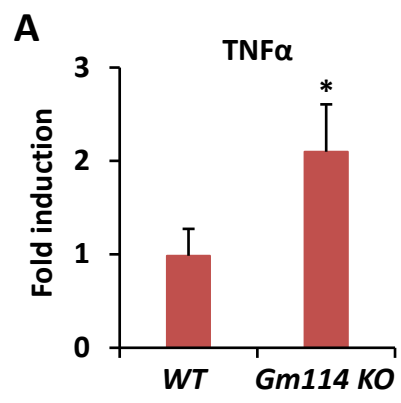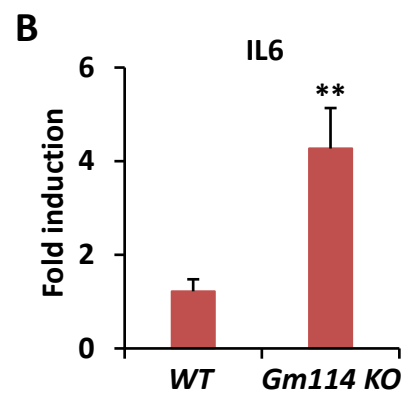

Supplement: Figure S2 — (A and B) 20-week WT and Gm114 KO mice were sacrificed. Intestines were dissected for RT-qPCR assays to monitor the mRNA levels of TNF α (A) and IL6 (B). Error bars represent SD (n = 3). The two-tailed Student’s t test was used to analyze statistical significance. * p < 0.05, ** p < 0.01. [file peerj-10-14145-s002.pdf]

## Figure S2

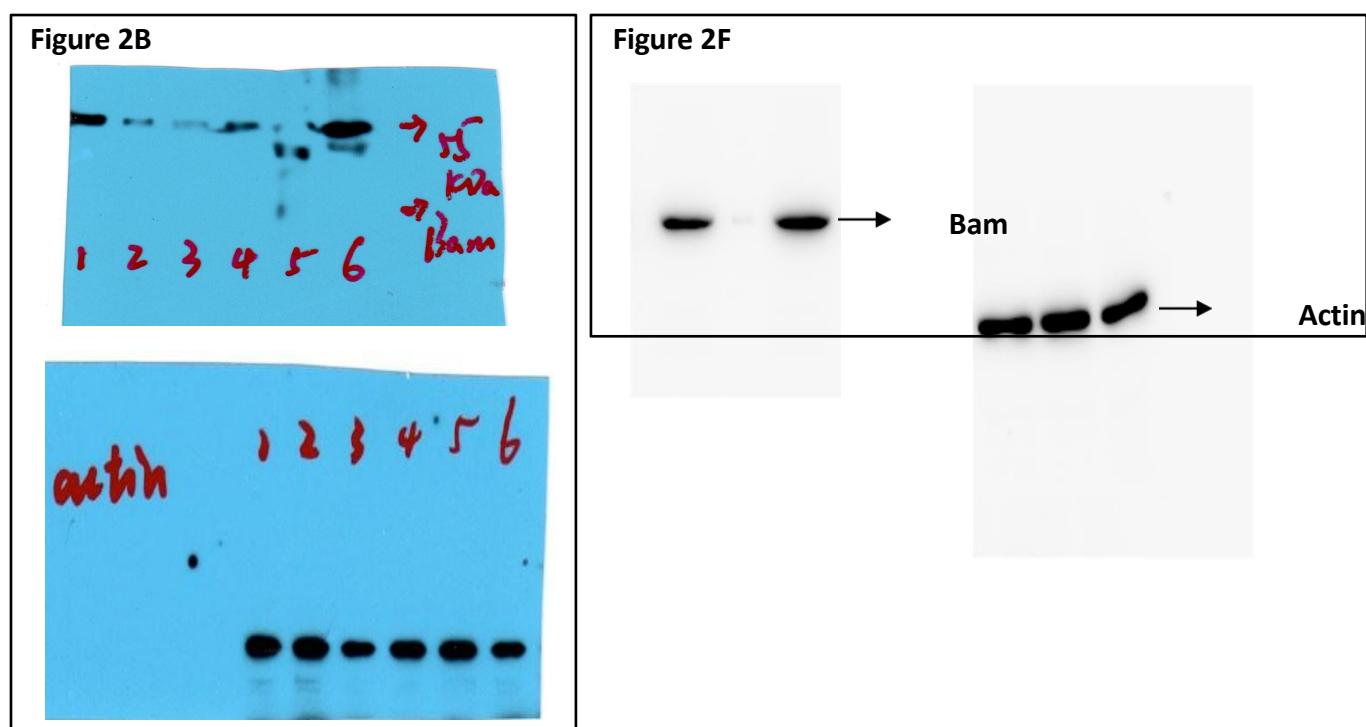

Figure S2. Original western blots in the main Figure 2B and 2F.

Supplement: Supplemental Information 4 [file peerj-10-14145-s004.pdf]
